# Bovine Papillomavirus Clastogenic Effect Analyzed in Comet Assay

**DOI:** 10.1155/2013/630683

**Published:** 2013-07-15

**Authors:** R. P. Araldi, T. C. Melo, N. Diniz, J. Mazzuchelli-de-Souza, R. F. Carvalho, W. Beçak, R. C. Stocco

**Affiliations:** ^1^Laboratório de Genética, Instituto Butantan, Avenida Vital Brasil, 1500, Butantã, 05503-900 São Paulo, SP, Brazil; ^2^Programa de Pós-graduação Interunidades em Biotecnologia, Instituto de Ciências Biomédicas, Universidade de São Paulo, Avenida Prof. Lineu Prestes, 2415 Edifício ICB-III-Cidade Universitária, 05508-900 São Paulo, SP, Brazil; ^3^Programa de Pós-graduação em Biologia Estrutural e Funcional, Universidade Federal de São Paulo, Rua Botucatu, 740, 04023-900 São Paulo, SP, Brazil; ^4^Departamento de Biologia, Universidade Federal da Integração Latino-Americana (UNILA), Avenida Tancredo Neves, 6731 bloco 4, 85867-970 Foz do Iguaçú, PR, Brazil

## Abstract

Bovine papillomavirus (BPV) is an oncogenic virus related to serious livestock diseases. Oncoproteins encoded by BPV are involved in several steps of cellular transformation and have been reported as presenting clastogenic effects in peripheral lymphocytes and primary culture cells. The aim of this study was to evaluate the clastogenic potential of BPV types 1, 2, and 4 by comet assay. Peripheral blood was collected from 37 bovines, 32 infected with different levels of papillomatosis (12 animals have no affection) and five calves, virus free (negative control). The viral identification showed presence of more than one virus type in 59.375% of the infected animals. Comet assay was performed according to alkaline technique. The Kruskal-Wallis test showed statistical difference between the negative control group and infected animals (P = 0.0015). The Dunn post hoc test showed difference comparing the infected animals with calves. Mann-Whitney *U* test verified no difference between animals infected with only one viral type and animals presenting more than one viral type. The comet assay is considered an efficient tool for assessment of damage in the host chromatin due to viral action, specifically highlighting viral activity in blood cells.

## 1. Introduction

 Bovine papillomavirus (BPV) is a widespread oncogenic virus found worldwide belonging to the Papillomaviridae family, which displays tropism for squamous epithelial and mucosal tissues. These viruses are associated with benign and malignant epithelial lesions. Specifically, BPV presents a double-stranded circular DNA, not coiled, with approximately 8 kb, surrounded by an icosahedral capsid consisting of 360 copies of the L1 protein of 55 kDa, 72 capsomeres arranged in approximately 12 copies of the L2 protein, 39 kDa [1–8]. The papillomavirus genome is divided into three regions: early, late, and noncoding long control region (LCR), separated by two polyadenylation sites [3]. The early control region occupies 50% of the viral genome and encodes E1, E2, E3, E4, E5, E6, and E7 proteins. The late control region occupies 40% of the genome and contains the genes that codify L1 and L2 capsid proteins and LCR, which comprises 10% of the genome, with 850 bp. However, it also contains the origin of replication and the binding sites of multiple transcription factors [3]. Oncoproteins encoded by BPV are involved in several steps of the cell transformation [1, 9]. 

In cattle, the correlation between papillomavirus and cancer has been investigated in view of the economic costs generated by viral infection [1, 8, 10, 11]. BPV is the etiological agent of bovine papillomatosis, infectious disease, characterized by the presence of hyperproliferative skin lesions (papillomas), causing significant economic loss to livestock ranchers and can progress to cancer with the action of cofactors [8, 10, 12]. 

 Currently, there are 13 BPV virus types described in the literature, although this number may be greater than twenty [7, 9]. The virus types are divided into three genres: *Deltapapillomavirus *(BPV-1, -2, and -13), *Epsilonpapillomavirus* (BPV-5 and -8), and *Xipapillomavirus* (BPV-3, -4, -6, -8, -9, -10, and -12), as well as the BPV-7 that remains not ranked in any genre [7]. Beside these, there are 16 new putative BPVs (BAA-1 to -4, BAPV-2 to -5, BAPV-7 to -10, BAPPV11MY and BPV/BR-UEL-2 to -5) [13]. According to Zhu et al. [13], BAA1 was detected in tongue epithelial papilloma, being designated BPV-12, and BPV/BR-UEL-4 described in ear cutaneous lesions was designated BPV-13 [9]. 

 According to Stocco dos Santos et al. [1], papillomavirus can act on host chromatin causing cytogenetic alterations, such as changes in ploidy, chromatin gaps and breaks, dicentric chromosomes and rings. Significant increase of chromosomal aberrations was detected in animals infected with BPV, affecting genomic stability [4]. However, to date, there are no studies evaluating the BPV clastogenic potential in peripheral blood cells analyzed by comet assay. 

 Comet assay or single cell gel electrophoresis was introduced by Östling and Johanson [14] and later modified by Singh et al. [15]. The comet assay is a simple and versatile technique that requires few eukaryotic cells, as well as having a vast DNA damage spectrum detection capacity [16–19]. In the test, cells are engulfed by agarose gel and spread over the slide, and then subjected to an electric field that promotes free DNA fragment migration, with the appearance of a comet [15]. The nuclear region causes the head of the comet to fragment, and the length of the tail is directly related to the intensity of the damage.

 The objective of this work was to evaluate the clastogenic potential of BPV types 1, 2, and 4 through comet assay in infected animals presenting cutaneous papillomatosis symptoms (hyperproliferative lesions-papillomas), asymptomatic (without papillomas) and calves, not infected, as negative control. The efficacy of the comet assay in clastogenic evaluations justifies this study.

## 2. Material and Methods

### 2.1. Ethics Statements

The protocols used in this study was approved by the Ethical Committee in Research of the Universidade Federal de São Paulo (Protocol number 1829/09) assigned by the President of this committee. All efforts were made to minimize any suffering in the animals.

### 2.2. BPV Diagnosis

#### 2.2.1. Animal Selection

37 bovines (*Bost aurus*) were selected: 32 adults, 12 asymptomatic (without visible cutaneous papillomas) and 20 symptomatic (with visible cutaneous papillomas) and 5 newborn calves that were separated from their mother after birth and did not receive breast milk. The presence of papillomas in others organs was not evaluated due the absence of clinical characteristics that suggest bladder and/or esophageal cancer. The farm does not have the presence of bracken fern *Pteridium aquilinum* that is involved in oncogenic and mutagenic processes in function of quercetin presence [20]. These calves were isolated in order to obtain virus negative controls. *Blood sample.* The peripheral blood samples were collected using the EDTA vacutainer. *Blood DNA extraction.* The extraction of DNA from peripheral blood cells was performed using the GenomicPrep Blood Mini Kit Illustra Spin (GE Healthcare, Buckinghamshire, UK), which uses enzymatic digestion method with protease K, according to the manufacturer. The quality of obtained DNA was verified using PCR by amplifying a bovine *β*-globin gene fragment [21]. 

#### 2.2.2. Viral Identification

Viral identification was performed using specific primers for BPV-1 (forward: 5′-GGAGCGCCTGCTAACTATAGGA-3′ and reverse: 5′-ATCTGTTGTTTGGGTGGTGAC-3′), which amplifies the L1 gene, resulting in a 301 bp amplicon, BPV-2 (forward: 5′-GTTATACCACCCAAAGAAGACCCT-3′ and reverse: 5′-CTGGTTGCAAÇAGCTCTCTTTCTC-3′), which amplifies the L2 gene, resulting in a 164 amplicon, and BPV-4 (forward; 5′-GCTGACCTTCCAGTCTTAAT-3′ and reverse; 5′-CAGTTTCAATCTCCTCTTCA-3′), which amplifies the E7 gene, resulting in a 170 bp amplicon. We choose these primers because the virus types are often observed in Brazil and in function of the association with esophageal (BPV-4) and bladder cancer (BPV-1 and -2). In detail, the amplification reactions were performed in a Veriti 96-well thermal cycler (Applied Biosystems, Singapore), with Master Mix (Promega, Madison, USA), under the following conditions: 5 minutes at 95°C, followed by 35 cycles of 1 minute and 30 seconds at 98°C, 2 minutes at 52°C, and 1 minute and 30 seconds at 72°C and a final extension step of 5 minutes at 72°C, for *β*-globin and specific primers. The PCR products were analyzed in 2% agarose gel electrophoresis stained with GelRed in TAE buffer, visualized under UV light. The images were captured through the software Kodak 1D 3.6.5. Cloned BPV-1, -2 and -4 genomes in *Escherichia coli *D5H*α* were used as positive controls. These clones form part of the biological collection of Genetic Laboratory of Butantan Institute. The fragments were purified using Illustra GFX PCR DNA and Gel Band Purification Kit (GE Healthcare, Buckinghamshire, UK). DNA concentration and purity were determined in a spectrophotometer BioPhotometer plus (Eppendorf, Hamburg, Germany) and submitted to sequencing reactions. *Sequencing*. The purified amplified products were sequenced in an ABI377 PRISM Genetic Analyzer. The quality of DNA sequences was checked, the overlapping fragments were assembled using the BioEdit package software BioEdit package 7.0.9.0 [22], and the nucleotide sequences were compared through BLAST (http://blast.ncbi.nlm.nih.gov/Blast.cgi).

### 2.3. Comet Assay

Comet assay was performed according to alkaline technique [15]. An aliquot of 10 *μ*L (0.1 × 10^5^ cells) was transferred to 0.2 mL polypropylene tubes and mixed with 75 *μ*L of low-melting-point agarose (0.7% in PBS) at 37°C, LMA optimum concentration (≤0.8%), without affecting the DNA migration [17], which was spread onto 76 × 26 mm microscope slides precoated with normal-melting-point agarose (1.5% in PBS) at 60°C and dried at 22°C overnight. After the agarose had solidified (4°C for 10 min), the coverslips were carefully removed, and the slides were immersed in lysis solution (2.5 M NaCl, 100 mM Na_2_EDTA, 10 mM Tris-HCl; pH 10; 1% Triton X-100, and 10% DMSO) for 1 hour at 4°C and then placed into a horizontal electrophoresis apparatus filled with buffer (1 mM Na_2_EDTA, 300 mM NaOH) at 4°C. The slides were incubated for 40 min in this buffer to unwind the DNA. The electrophoresis was run for 20 min at fixed voltage of 25 V (0.83 V/cm) and 300 mA. At the end of electrophoresis, the slides were washed three times with neutralization buffer (0.4 M Tris-HCl, pH 7.5) and once in 100% ethanol. All steps described above were carried out with the lights out to avoid DNA damage. The slides were stained with 20 *μ*L of propidium iodide (4 *μ*g/mL) and visualized in Carl Zeiss Axio Scope A1 fluorescent microscopy. Nucleoids were classified from 0 (without lesion) to 2 (major damage), and the number of nucleoids observed per class was multiplied to the class value, resulting in a comet score. *Statistical analysis. *The data were analyzed using the Kruskal-Wallis test, followed by post hoc Dunn test and Mann-Whitney *U* test, both with significance level of 5%, through BioEstat 5.3. software.

## 3. Results

### 3.1. Viral Genotyping

**Figure 1:**
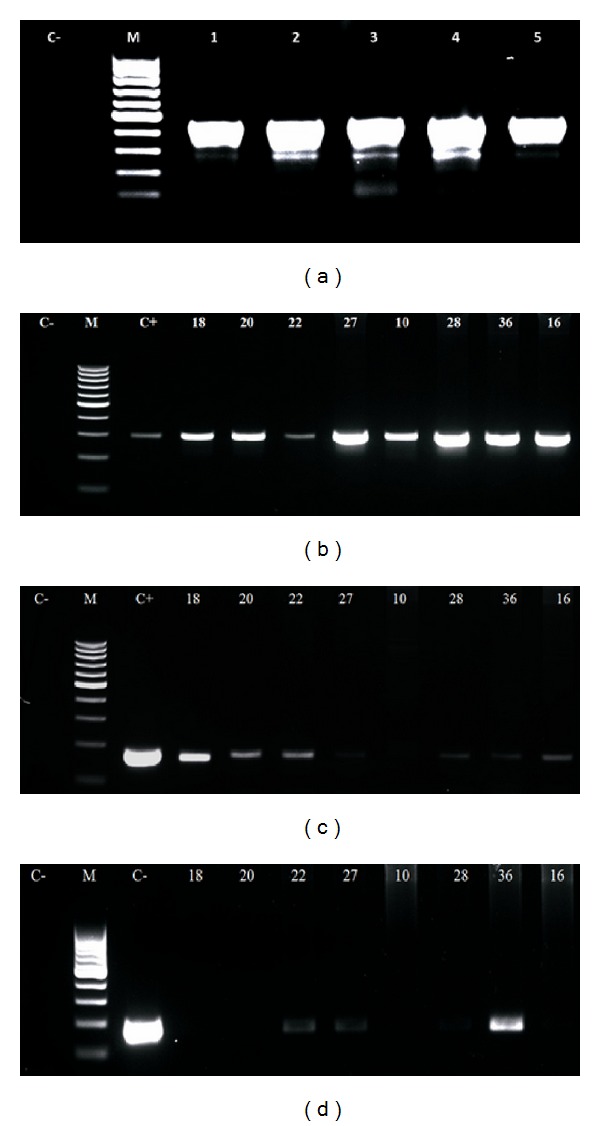
Electrophoresis's images of: (a) (*β*-globin amplification, resulting in an amplicon of 450 bp), (b) (amplification of L1 gene, using primer to BPV-1, showing an amplicon of 301 bp), (c) (amplification of L2 gene, using primer to BPV-2, showing an amplicon of 164 bp) and (d) (amplification of E7 gene, using primer to BPV-4, showing an amplicon of 170 bp), being C− (negative control) and C+ (positive control).

PCR using specific primers to *β*-globin revealed DNA quality enough for further PCR procedures: all samples resulted in a 450 bp amplicon. We selected the specific primers for BPV-1, -2, and -4 due to their prevalence in the herd, and we could detect the virus sequences in peripheral blood cells collected from the adult animals, with and without skin papillomas (Figure 1(a)). The resulting bands were purified and sequenced to confirm the genotyping of the amplification products. The sequences were aligned through the BioEdit 7.0.9.0, and the nucleotide comparison was done through the BLAST tool, confirming the specificity of the primers employed. Using these primers, coinfection was reported in 59.375% of the infected bovines—19 animals (Table 1(a)). The use of these primers did not detect the presence of BPV in calf peripheral blood samples, with this group being considered a negative control.

### 3.2. Comet Assay

**Figure 2:**
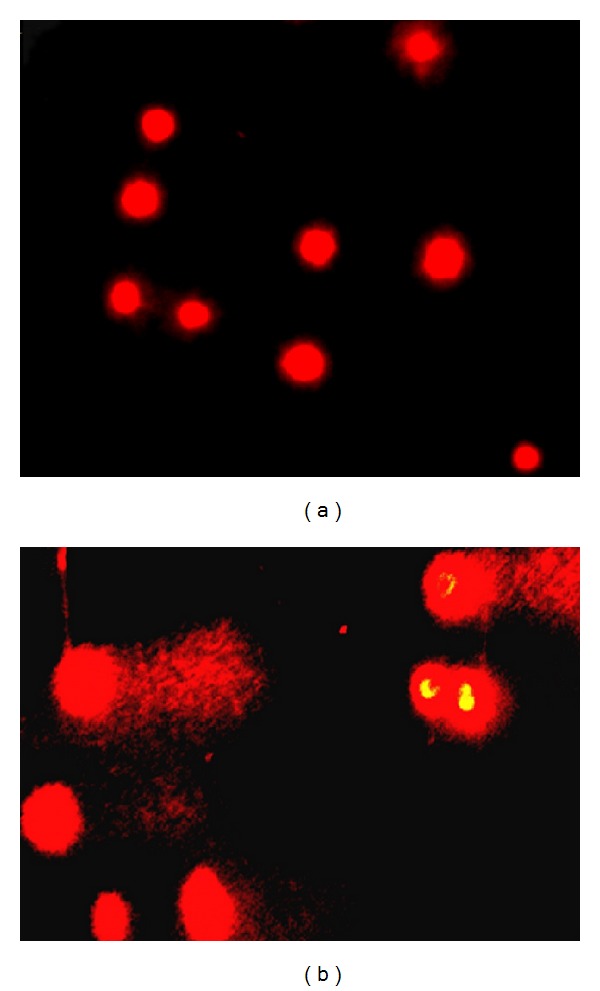
(a) Images of class 0 nucleoids, observed in calves (negative control) and (b) class 2 nucleoids, observed in symptomatic adult bovine, showing DNA fragmentation that is indicative of clastogenicity.

The samples of peripheral blood cells collected from infected animals and calves (negative control) were evaluated through comet assay, counting 100 nucleoids per sample that were evaluated and classified as 0 (without damage), 1 (medium damage), and 2 (maximum damage) according to Figure 2. The nucleoid value per class was multiplied by the respective class value, resulting in the comet score (Table 1(a)).

Based on these data, a Kruskal-Wallis test was used, with 5% significance level, through BioEstat 5.3. software to compare the different groups (infected and not infected group). The test reveled statistical differences between the groups (*H* = 12.9714 and *P*  value = 0.0015). The Dunn post hoc test showed difference in score values among calf and infected animals (asymptomatic and symptomatic) (Table 1(b)). Mann-Whitney *U* test was done using the same software to compare the level of clastogenicity between animals, which showed just one virus type, and coinfected cattle. The results did not reveal statistical differences between animals infected with only one viral type and animals presenting more than one viral type (Table 1(c)).

## 4. Discussion

The carcinogenic mechanisms related to BPV are not yet fully elucidated; however, it is known that the malignancy is caused by mutations induced by the virus, associated with the action of viral protein E6, which accelerates the degradation of p53 and E7, which degrades the tumor suppressor protein pRb. These processes change the transcriptional pathway through degradation of transcription factors, activating telomerase, affecting the DNA repair system, and leading to an increase of damage in host genetic material [6, 23–25]. According to You [6], the protein E7 interacts with microtubules in mitosis, causing defects in the alignment of chromosomes during pre-metaphase, resulting in cytogenetic alteration [1, 26]. Wade et al. [25] discussed that the BPV oncoproteins can act on the signal transduction, allowing the return of interphase spinous epithelial cells to synthesis phase, resulting in mutated cell proliferation. Oncoproteins E6 and E7 induce immortalization of transformed cells [3]. According to Primrose and Twyman [27], these oncoproteins are required in the process of viral replication and act in the process of oncogenic transformation of the host cell. Furthermore, the accelerated p53 proteasomal degradation has a prominent role in the carcinogenic action of the virus, since the p53 protein function is to check the integrity of the genome, preventing the proliferation of mutated cells. The p53 accumulates in the cell nucleus, keeping the mitotic cycle in early G1 phase by activating p21 gene, whose gene product inhibits the action of cycling-dependent kinases (CDKs) and activates genes related to repair system [28].

Melo et al. [26] analyzed peripheral blood lymphocytes obtained from clinically asymptomatic bovines and showed a high level of chromosomal aberrations, suggesting the BPV action in the host chromatin even before the clinical manifestation of papillomatosis. These data also indicate that the skin lesions (papillomas) represent visible clinical manifestations, but the virus or its DNA sequences, detected in peripheral blood, can represent a potential risk to carcinogenesis.

In this study, we verified BPV clastogenic action by comet assay indicating chromatin instability. The comet assay results demonstrated that the BPV is able to induce severe DNA damages, which hinder the repair system, this is because the assay allows to evaluate the DNA double-strand breaks (DSBs) and critical lesions that are involved in genomic instability [29–31].

The DSB is associated with the homologous recombination from the formation of DNA simple strand that invades the template strand, originating a Holliday junction, which migrates to the resolution of heteroduplex [29, 31]. However, unrepaired DSBs leave to apoptosis or cell-cycle arrest, resulting in carcinogenesis [29]. There are lines of evidence that unrepaired DSBs could leave to telomeric breaks and fusion events, also associated with oncogenic process [29, 31].

The viral oncoproteins affect the repair system, allowing an accumulation of stochastic mutations and resulting in increased genomic instability [17]. According to Duensing and Münger [23], breaks in DNA affect the cell-cycle checkpoint that is associated with genomic instability, leading to hyper proliferation, featuring an oncogenic process. According to the European Study Group on Health's Biomarkers, cytogenetic findings, as a high frequency of chromosomal aberrations, including breaks in single or double-stranded, are associated to the carcinogenesis [32]. So, at the same time the presence of BPV is causing DSBs, the virus affects the repair system, favoring oncogenic process associated with unrepaired DSBs.

The virus presence in the blood can suggest one alternative pathway to infection, in which asymptomatic but infected cattle could turn symptomatic from a tissue injury, considering that a lesion causes an inflammatory process with lymphocyte infiltration [33]. The presence of BPV in leukocytes was demonstrated, had been observed the BPV presence in peripheral blood mononuclear cells [33] and the presence of L1 protein in CD4+ and CD8+ leukocytes, representing a potential infection sites to BPV-2 [34]. The possibility of the existence of endogenous pathway of infection has been discussed by Wobeser et al. [33], who suggested that the mononuclear cells act as a source of infection for inflammation sites, as inflamed areas become more susceptible to infection by BPV. Furthermore, it is known that lymphocytes express heparan sulfate, being cells susceptible to infection by papillomavirus [33]. Another observation that supports the possibility of infection was reported by Hartl et al. [35], who found that the spontaneous regression of papillomavirus in transient infections in humans and cattle is accompanied by an accumulation of active lymphocytes CD4+ and CD8+. So, in this pathway, the infiltration of BPV infected cell could develop a tumorigenic process from a clonal evolution started in a histologically normal tissue [35, 36].

## 5. Conclusion

This study presents direct evidence of DNA damage related to bovine papillomavirus in blood cells, indicating a viral activity in peripheral blood. The levels of damage were analyzed in order to verify if the presence of more than one viral type could increase the clastogenic viral action, but no significant differences could be detected. The results showed the same DNA damage both in presence or absence of cutaneous papillomas, indicating that the presence of bovine papillomatosis just represents clinical symptoms due the BPV presence; however, the BPV presence in peripheral blood leaves to double-stranded breaks, which is associated to carcinogenesis, affecting the healthy animal, as previously reported [1, 26]. Comet assay can be discussed as an interesting technique to evaluate DNA damage which, in this special situation, is related to viral action, demonstrating viral activity in different sites as blood cells.

## Figures and Tables

**Table 1a:** (a)

Controls	Clinical	Virus type	Class of nucleoids			Score
			0	1	2	
1	Asymptomatic	—	93	4	3	10
2	Asymptomatic	—	92	5	3	11
3	Asymptomatic	—	96	1	3	7
4	Asymptomatic	—	90	8	2	12
5	Asymptomatic	—	95	3	2	7

Infected bovines						

6	Asymptomatic	BPV-1	15	60	25	110
7	Asymptomatic	BPV-2	57	36	7	50
8	Asymptomatic	BPV-1	75	19	6	31
9	Asymptomatic	BPV-1, -2	43	17	40	97
10	Asymptomatic	BPV-1	73	19	8	35
11	Asymptomatic	BPV-1, -2	47	26	27	80
12	Asymptomatic	BPV-1	38	26	36	98
13	Asymptomatic	BPV-1, -2	62	13	25	63
14	Asymptomatic	BPV-2	72	21	7	35
15	Asymptomatic	BPV-1, -2	63	12	25	62
16	Asymptomatic	BPV-1, -2	58	8	34	76
17	Asymptomatic	BPV-1, -2	40	42	18	78
18	Symptomatic	BPV-1, -2	61	30	9	48
19	Symptomatic	BPV-2	22	13	65	143
20	Symptomatic	BPV-1, -2	65	12	23	58
21	Symptomatic	BPV-1, -2	57	21	22	65
22	Symptomatic	BPV-1, -2, -4	31	31	38	107
23	Symptomatic	BPV-1	89	6	5	16
24	Symptomatic	BPV-1	92	2	6	14
25	Symptomatic	BPV-1	69	25	6	37
26	Symptomatic	BPV-1	41	10	49	108
27	Symptomatic	BPV-1, -2, -4	70	21	9	39
28	Symptomatic	BPV-1, -2, -4	65	26	9	44
29	Symptomatic	BPV-2	87	8	5	18
30	Symptomatic	BPV-1, -2	0	66	34	134
31	Symptomatic	BPV-1, -2	60	31	9	49
32	Symptomatic	BPV-2	52	23	25	73
33	Symptomatic	BPV-1, -2	72	9	19	47
34	Symptomatic	BPV-1, -2	91	7	2	11
35	Symptomatic	BPV-1, -2	74	15	11	37
36	Symptomatic	BPV1, -2, -4	82	13	5	23
37	Symptomatic	BPV-1, -2	36	22	42	106

**Table 1b:** (b)

Groups	Average post	*Z* calculated	*P* value
Calves and asymptomatic	3.300	3.5348	<0.05
Calves and symptomatic	23.666	3.1097	<0.05
Asymptomatic and symptomatic	20.125	0.8961	n.s.

*H* = 12.9714, *P* = 0.0015, *R* of calves = 16.5, *R* of asymptomatic = 284.0 and *R* of symptomatic = 402.5.

**Table 1c:** (c)

Monoinfected sample	Score	Coinfected sample	Score
6	110	9	97
7	50	11	80
8	31	13	63
10	35	15	62
12	98	16	76
14	35	17	78
19	143	18	48
23	16	20	20
24	14	21	21
25	37	22	22
26	108	27	39
29	18	28	44
32	74	30	134
—	—	31	49
—	—	33	47
—	—	34	11
—	—	35	37
—	—	36	23
—	—	37	106

Mann-Whitney *U* test results: *U* = 102.5, *Z*(*U*) = 0.80, *P* value = 0.21.
